# Shiga toxin 2 from enterohemorrhagic *Escherichia coli* induces reactive glial cells and neurovascular disarrangements including edema and lipid peroxidation in the murine brain hippocampus

**DOI:** 10.1186/s12929-019-0509-x

**Published:** 2019-02-07

**Authors:** Clara Berdasco, Alipio Pinto, Valeria Calabró, David Arenas, Adriana Cangelosi, Patricia Geoghegan, Pablo Evelson, Jorge Goldstein

**Affiliations:** 1 Universidad de Buenos Aires, Consejo Nacional de Investigaciones Científicas y Técnicas (CONICET), Instituto de Fisiología y Biofísica ‘‘Houssay” (IFIBIO), Laboratorio de Neurofisiopatología, Facultad de Medicina, Paraguay 2155 piso 7, 1121 Buenos Aires, Argentina; 2 Universidad de Buenos Aires, CONICET, Instituto de Bioquímica y Medicina Molecular (IBIMOL), Facultad de Farmacia y Bioquímica, Buenos Aires, Argentina; 3 Centro Nacional de Control de Calidad de Biológicos (CNCCB), ANLIS “Dr. Carlos G. Malbrán”, Ciudad Autónoma de Buenos Aires, Argentina; 4 Universidad de Buenos Aires, Facultad de Farmacia y Bioquímica, Departamento de Química Analítica y Fisicoquímica, Cátedra de Química General e Inorgánica, Buenos Aires, Argentina

**Keywords:** Encephalopathy, HUS, Inflammation, Lipid peroxidation, Hippocampus

## Abstract

**Background:**

Shiga toxin 2 from enterohemorrhagic *Escherichia coli* is the etiologic agent of bloody diarrhea, hemolytic uremic syndrome and derived encephalopathies that may result to death in patients. Being a Gram negative bacterium, lipopolysaccharide is also released. Particularly, the hippocampus has been found affected in patients intoxicated with Shiga toxin 2. In the current work, the deleterious effects of Shiga toxin 2 and lipopolysaccharide are investigated in detail in hippocampal cells for the first time in a translational murine model, providing conclusive evidences on how these toxins may damage in the observed clinic cases.

**Methods:**

Male NIH mice (25 g) were injected intravenously with saline solution, lipopolysaccharide, Shiga toxin 2 or a combination of Shiga toxin 2 with lipopolysaccharide. Brain water content assay was made to determine brain edema. Another set of animals were intracardially perfused with a fixative solution and their brains were subjected to immunofluorescence with lectins to determine the microvasculature profile, and anti-GFAP, anti-NeuN, anti-MBP and anti-Iba1 to study reactive astrocytes, neuronal damage, myelin dysarrangements and microglial state respectively. Finally, the Thiobarbituric Acid Reactive Substances Assay was made to determine lipid peroxidation. In all assays, statistical significance was performed using the One-way analysis of variance followed by Bonferroni post hoc test.

**Results:**

Systemic sublethal administration of Shiga toxin 2 increased the expressions of astrocytic GFAP and microglial Iba1, and decreased the expressions of endothelial glycocalyx, NeuN neurons from CA1 pyramidal layer and oligodendrocytic MBP myelin sheath from the fimbria of the hippocampus. In addition, increased interstitial fluids and Thiobarbituric Acid Reactive Substances-derived lipid peroxidation were also found. The observed outcomes were enhanced when sublethal administration of Shiga toxin 2 was co-administered together with lipopolysaccharide.

**Conclusion:**

Systemic sublethal administration of Shiga toxin 2 produced a deterioration of the cells that integrate the vascular unit displaying astrocytic and microglial reactive profiles, while edema and lipid peroxidation were also observed. The contribution of lipopolysaccharide to pathogenicity caused by Shiga toxin 2 resulted to enhance the observed hippocampal damage.

## Background

Hemolytic uremic syndrome (HUS) is an illness characterized by a triad of events that include nonimmune haemolytic anaemia, thrombocytopenia and acute renal failure caused by Shiga toxin 2 (Stx2) from enterohemorrhagic *Escherichia coli* (EHEC). Patients may initially develop bloody diarrhea when EHEC succeeds to colonize the gastrointestinal tract. Once Stx2 reaches the circulation it may target endothelial, kidney and/or brain cells through the Stx2 globotriaosylceramide receptor (Gb3) causing cytotoxicity [1]. Neurological impairment frequently occurs and is associated with a worse prognostic [2]. Besides Stx2 pathogenicity, lipopolysaccharide (LPS) is another virulence factor that is also released form EHEC [3], for being a Gram negative bacteria. The action of LPS has been described as an enhancer of the deleterious effects of Stx2 in different cells [4] and organs such as the brain [4, 5].

Particularly in Argentina, post diarrheal HUS is endemic and possesses the highest rate of pediatric cases in the planet. 400 cases are reported annually with an incidence of 10 to 17 cases per 100,000 children under 5 years old, and among them lethality was reported between 1 and 4% [6]. In addition, a high percent of the pediatric patients with HUS develop central nervous system (CNS) dysfunctions [2]. As the grade of severity in HUS cases is usually associated with neurological dysfunctions, the mortality rate rises significantly between 2 to 3-folds when the CNS is involved [7]. Moreover, it has been reported that about 9 to 15% showed neurological symptoms before the onset of HUS [7].

Although reports on cognitive dysfunctions have been reported in HUS patients, research in brain cognitive areas such as the hippocampus has been scarcely described. As mentioned, a case of cognitive dysfunctions in HUS patients occurred during a large outbreak of diarrhea-associated HUS in Germany 2011 [8]. The responsible was an unusual enteroaggregative Shiga toxin-producing *Escherichia coli* (STEC) O104:H4. 2987 adults were registered with gastrointestinal infections. 22% of them underwent HUS, while more than a half of them acquired neurologic alterations and about 58% of those with neurologic involvement suffered from cognitive dysfunction, like trouble finding words, severe alteration of consciousness or late memory decline [9]. In addition, other reports of Stx2-derived encephalopathy observed alteration of memory and consciousness, seizures and coma [10]. As known, the hippocampus is involved in cognitive functions including memory formation [11]. Functionally, the hippocampus is one of the brain areas found vulnerable to the deleterious actions of Stx2 [8]. Neurons from the hippocampal CA1 region are mainly involved in memory tasks. Among them long-term potentiation and spatial learning is essentially controlled by these neurons [12]. As Stx2 caused memory alterations including orientation deficits in patients [13], and as we demonstrated the presence of the Stx2-Gb3 cell receptor in neurons from the CA1 layer [14], we prompted us to study particularly the cellular events that occurred in such and surroundings layers of the hippocampus. In the current work, the deleterious effects of Stx2 and LPS are investigated in detail in hippocampal cells for the first time, providing conclusive evidences on how these toxins may damage in the observed clinic cases.

## Methods

### Animals

NIH male Swiss mice of approximately 25 g (30 days, housed under 12 h-light/12 h-dark cycles) were divided into 4 different groups according to their intravenous (i.v.) treatment: control (saline solution), LPS (800 ng, *E. coli* 055:B5, Sigma, St. Louis, MO, USA), Stx2 (0.5 ng or 1 ng, Phoenix Laboratory, Tufts Medical Center, Boston, MA, USA) and Stx2 + LPS (1 ng and 800 ng ± respectively). The total amount of i.v. solution injected was 100 μl per mouse and the Stx2 dose was about 60% of the LD_50_ (1.6 ng per mice). Food and water were provided ad libitum and the experimental protocols and euthanasia procedures were reviewed and approved by the Institutional Animal Care and Use Committee of the School of Medicine of Universidad de Buenos Aires, Argentina (Resolution N° 046/2017). All the procedures were performed in accordance with the EEC guidelines for care and use of experimental animals (EEC Council 86/609).

### Brain water content assay

The dry/wet weight method was applied to the brain of mice divided in groups according to their i.v. treatments as described above. The mice (*n* = 10) were sacrificed at 6, 12, 24 or 48 h post-i.v. treatment. The entire brain was used for this assay and they were weighted on a precision balance of 10 μg accuracy (Acculab ALC-110.4, Brooklyn, NY, USA) to obtain their wet weight. Then, they were dried in an incubator at 56 °C for 6 days to obtain their dry weight. The water content was determined from the difference between the wet weight and dry weight, according to Testylier et al. (2007) [18].

### Histo and immunofluorescence assay

Mice (*n* = 4 for each treatment) were anesthetized with pentobarbital (100 mg/kg) and intracardiacally perfused with paraformaldehyde 4% diluted in phosphate buffer saline (PBS) 0.1 M, pH 7.4; at 2, 4, 7 and 20 days. Day 0 was set as the day of the i.v. treatment. The brains were removed from the skulls and post-fixed overnight at 4 °C with the same described fixative solution, then daily cryopreserved with increasing concentrations of sucrose diluted in PBS (10, 20 and 30%). Brain sections of 20 μm were cut in a cryostat. The brains slices were storage at − 20 °C in a cryopreservant solution (50% PBS, 30% ethylene glycol and 20% glycerol) until the day of histo and/or immunofluorescence assay.

The histofluorescence assay to detect the glycocalyx microvasculature was made with 10 μg/ml of biotinylated lectin from *Lycopersicon esculentum* (Sigma, St. Louis, MO, USA). Brain slices were first washed several times with PBS 10 mM to be incubated overnight with lectin at 4 °C. The slices were then washed several times with PBS 10 mM and incubated overnight with streptavidine Alexa Fluor 488 (Invitrogen Molecular Probes, Carlsbad, California, USA) at 4 °C followed by several washed in PBS to be mounted on slides for epifluorescence (Olympus BX50, Miami, Fl, USA) and confocal microscope (Olympus FV1000, Miami, Fl, USA) studies.

For the immunofluorescence assay, after several rinses with PBS 10 mM, brain slices were incubated with 10% fetal goat serum blocking solution in PBS 10 mM (Sigma, St. Louis, MO, USA) and 1% Triton X-100 (Sigma, St. Louis, MO, USA) for 1 h. The sections were immediately incubated with the following primary antibody (with Triton X-100 at 0,3%): rabbit anti-GFAP (1:500 - Dako, 225 Glostrup, Denmark), mouse anti-NeuN (1:250 - Millipore, Temecula, CA, USA), rabbit anti-MBP (1:500 - Dako, Glostrup, Denmark), goat anti-Iba1 (1:250 - Millipore, Temecula, Ca, USA) and rat anti-Gb3 (CD77, 1:250 - Serotec, Kidington, UK) overnight at 4 °C to identify astrocytes, neurons, oligodendrocytes, microglial cells and the Stx2 Gb3 receptor respectively. Once the sections were rinsed several times with PBS 10 mM they were incubated with their respective secondary antibodies (with Triton X-100 at 0,3%): goat anti-rabbit Alexa Fluor 555 (1:500 - Invitrogen Molecular Probes, Carlsbad, California, USA), goat anti-mouse Alexa Fluor 555 (1:500 - Amersham, GE, Piscataway, NJ, USA), donkey anti-goat Alexa Fluor 555 (1:500 - Millipore, Temecula, Ca, USA) and goat anti-rat FITC (Jackson Immuno Research, West Grove, PA, USA) overnight at 4 °C. Finally, all hippocampal brain slices were incubated with Hoechst 33342 (1:500 - Sigma, St. Louis, MO, USA) for 15 min at room temperature to show the nuclei of brain cells. Negative controls were made by omitting the primary antibody. The hippocampal CA1 area was observed with an Olympus BX50 epifluorescence microscope provided with a Cool-Snap digital camera and Olympus FV1000 confocal. Micrographs were obtained from CA1 hippocampal field, (− 1.70 and − 1.82 mm from bregma). Analysis of lectin histofluorescence, GFAP and Iba1 immunofluorescences were made in hippocampal CA1 stratum radiatum (Rad), while immunofluorescence to neuronal NeuN was analyzed in the CA1 pyramidal layer (Py). Finally, immunofluorescence to MBP was observed in the hippocampal fimbria (fi). The obtained micrographs were analyzed with the Fiji ImageJ software (NIH, MD, USA). GFAP and Iba1 expression levels were measured as the integral optical density (IOD) per cell, while histofluorescence to lectin and MBP expression levels were measured as total IOD of each micrograph.

For this purpose, all images were opened on Fiji ImageJ, the color channels were splitted and the one with the specific color was selected. In order to select only the immunopositive cells, the threshold tool was employed and the IOD was measured with the Analyze>Measure tool, and the “mean” was selected. The criteria to determine endothelial damage on lectin images were the number of lectin positive particles and the area occupied by the microvessels. For this purpose, the images were calibrated with a pre-set scale bar, the straight tool was employed to set a line of the same size of the scale bar and using the set measurements the length of the line was calibrated. After that, the color channels were splitted, the green color was selected and the same steps to analyze the IOD were done, with the difference that in this case, the measurements of “area” and “count” were selected. Finally, the measurement in 3 different places of the Py was made to determine neuronal damage in the Py layer. For this purpose, the scale bar was set as it was described before, and the thickness of the pyramidal layer was measured using the straight tool.

### Thiobarbituric acid reactive substances assay

The malondialdehyde (MDA) content in hippocampus (*n* = 4) was evaluated as thiobarbituric acid reactive substances (TBARS) by a fluorometric assay with modifications at 12 h and 24 h following toxin treatments. Butylhydroxytoluene (4% *w*/*v* in ethanol) was used to prevent non-physiological TBARS formation during the sample processing [15]. Results are expressed as pmoles of MDA per mg of protein. The MDA standard was prepared from 1,1,3,3,-tetramethoxypropane.

### Statistical analysis

The data are presented as mean ± SEM. In all assays, the statistical significance were performed using the One-way analysis of variance (ANOVA) followed by Bonferroni post hoc test (GraphPad Prism 4, GraphPad Software Inc.) between the 4 i.v. treatments (control, LPS, Stx2 and Stx2 + LPS). The criterion for significance was *p* < 0.05 for all the experiments.

## Results

### Water content of murine brains was increased following systemic administration of a sublethal dose of Stx2

**Fig. 1:**
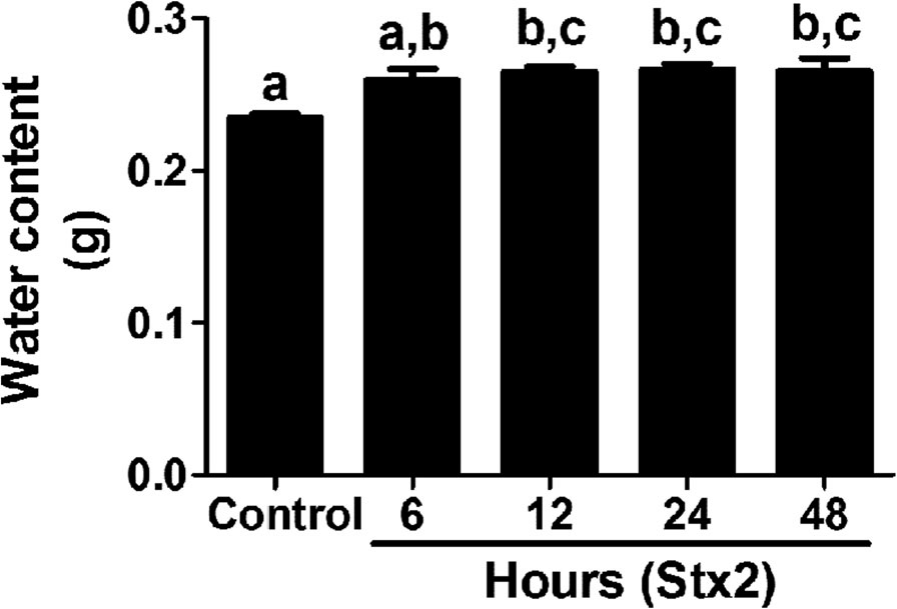
Stx2 increases the water content in murine brains. The letters above each column (a, b, c) mean significant differences. Data were analyzed by One-way ANOVA, Bonferroni’s post hoc test, *p* < 0.05, *n* = 10

In previous works sub-lethal dose of Stx2 was determined and administered to a characterized murine model of encephalopathy, to mimic and to unravel the cell mechanisms that may occur beyond the clinical signs observed in patients suffering from HUS [16]. Systemic administration of Stx2 provoked disarrangements on striatal blood brain barrier (BBB) cell components evidenced by immunofluorescence techniques [17] and perivascular edema was confirmed by electron microscopy [16]. In the present work, the brain water content was assessed following a characterized protocol [18] to determine whether the observed increase in reactive astrocytes (Fig. 3) and lectin bound to glycocalyx molecules derived to a decrease in the area occupied by these molecules in the BBB (Fig. 4) and whether these events correlated with the brain edema. As observed, a significant rise in water content was detected after 12 h of Stx2 administration. This rise was kept at 48 h (Fig. 1).

### Stx2 increased the expression levels of glial fibrilar acidic protein (GFAP) in the murine brain hippocampus in a dose dependent manner

GFAP is a cytoskeletal protein used as a specific marker to identify astrocytes in a reactive state because of a wide variety of brain injuries [19]. Two different sublethal doses of Stx2 were intravenously (i.v.) administered to determine whether deleterious effects of the toxin in brain cells may occur in a dose dependent manner (0.5 and 1 ng) and the day 4 was chosen to observe these effects. In our murine model, sublethal administration of Stx2 maximally induced reactive astrocytes and other events in the motor cortex and striatum at day 4 [5, 17]. At this context, the day 4 was initially chosen in order to establish the amount of Stx2 to be used in this work, and latter, a time curve was designed in order to determine the time Stx2 maximally induce reactive astrocytes but particularly in the murine hippocampal area CA1.

**Fig. 2:**
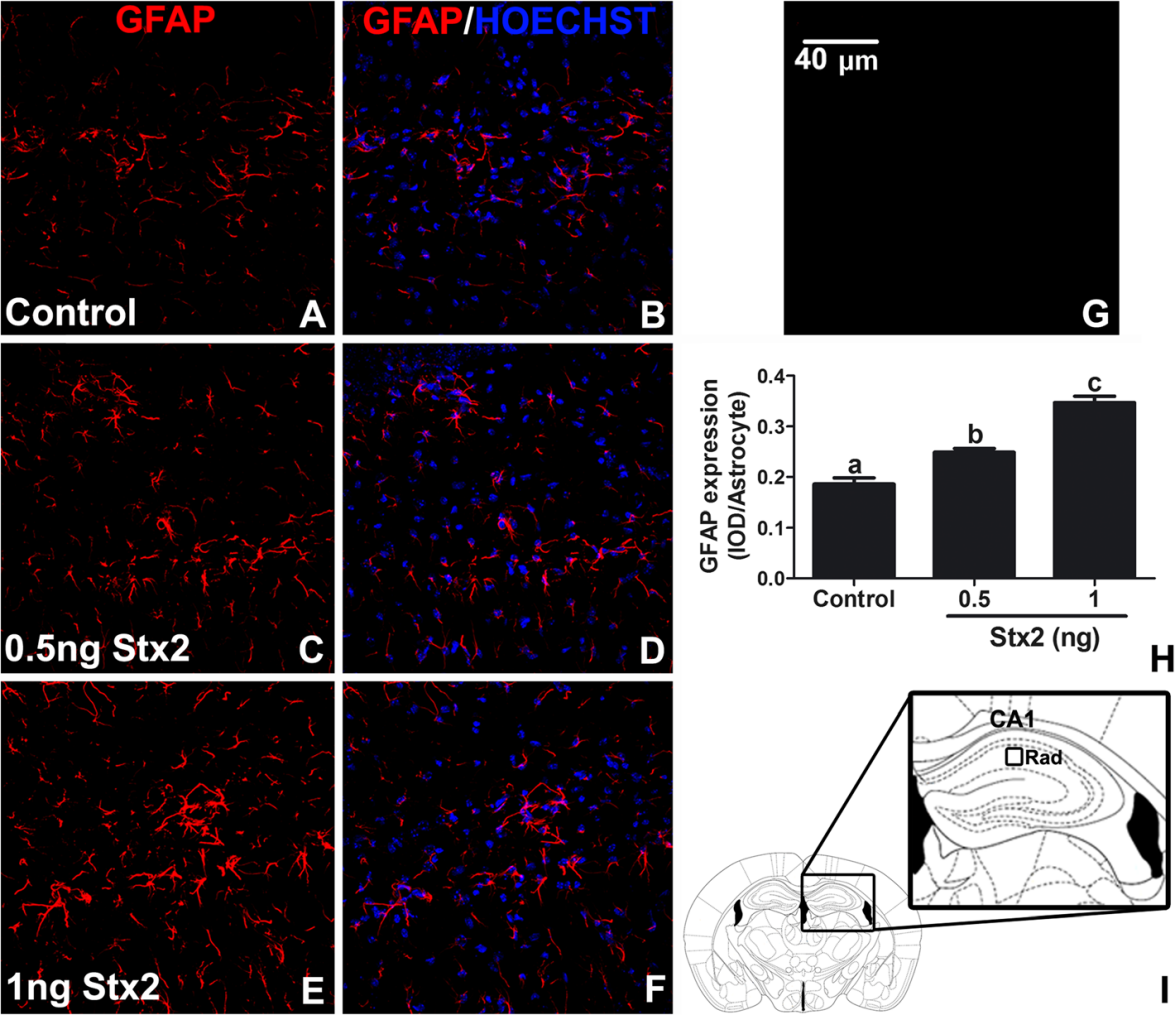
Stx2 produces reactive astrocytes in a dose dependent manner. Control (**a**-**b**); 0.5 ng of Stx2 (**c**-**d**); 1 ng of Stx2 (**e**-**f**) after 4 days of treatment. Immunofluorescence using an anti-GFAP antibody (**a**, **c**, **e**). Merge images between anti-GFAP immunofluorescence and Hoechst histofluorescence (**b**, **d**, **f**). Negative controls by omitting primary antibody (**g**). Quantification of integral optical density (IOD) from reactive astrocytes (**h**). The drawn square shows the area analyzed which corresponds to the hippocampal CA1 Rad area (**i**). Different letters (a, b, c) above the columns indicate significant differences between each dose (**h**). Scale bar in Fig. G applies to all micrographs. Data were analyzed by One-way ANOVA, Bonferroni’s post hoc test, *p* < 0.05, *n* = 4

A basal immuno-expression of GFAP was observed in astrocytes located in the hippocampal CA1 stratum radiatum (Fig. 2I) from control treated mice (Fig. 2a-b). In contrast to this, i.v. administration of 0.5 ng of Stx2 resulted to significantly increase GFAP immunoexpression (Fig. 2c-d). Moreover, i.v. administration of 1 ng of Stx2 (Fig. 2e-f) significantly increased even more reactive astrocytes (Fig. 2h). According to the preceding results, 1 ng was selected to study the deleterious effects of this toxin in the hippocampus. No immunofluorescence was observed in negative controls by omitting primary antibody (Fig. 2g).

### Systemic administration of Stx2 produced reactive astrocytes and the combination of this toxin with LPS resulted to enhance this effect

**Fig. 3:**
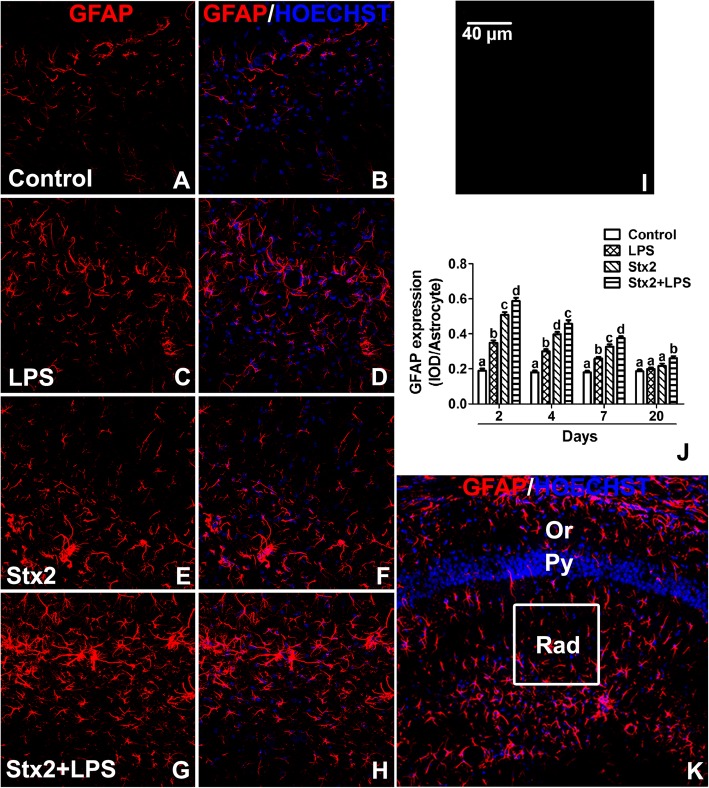
Stx2 and Stx2 + LPS produce reactive astrocytes. Representative micrographs of murine brain hippocampuses after 2 days of control, LPS, Stx2 or Stx2 + LPS treatments (A-H). Immunofluorescence using an anti-GFAP antibody (**a**, **c**, **e**, **g**). Merge images between anti-GFAP immunofluorescence and Hoechst histofluorescence (**b**, **d**, **f**, **h**, **k**). Control-treated mice (**a**-**b**); LPS-treated mice (**c**-**d**); Stx2-treated mice (**e**-**f**) and co-administration with Stx2 + LPS-treated mice (**g**-**h**). Negative control by omitting a primary antibody (**i**). Expression levels of GFAP from reactive astrocytes under all treatments at 2, 4, 7, and 20 days (**j**). A low magnification micrograph shows hippocampal layers: oriens layer (Or), pyramidal layer (Py), stratum radiatum (Rad) and the area analyzed in this study was Rad from the CA1 hippocampal area (the drawn square shows the area analyzed) (**k**). Different letters (a, b, c, d) above the columns indicate a significant difference between the 4 different i.v.-treated groups (**j**). The scale bar in Fig. I applies to all micrographs. Data were analyzed by One-way ANOVA, Bonferroni’s post hoc test, *p* < 0.05, *n* = 4

GFAP immunoexpression was measured in hippocampal stratum radiatum of the CA1 area (Fig. 3k) to determine whether systemic administration of Stx2 and/or LPS caused reactive astrocytes. According to this, maximal expression of GFAP was observed in all mice treated with both toxins at day 2 compared with the same treatments of the other 3 days (4, 7 and 20 days) as shown in Fig. 3. Co-administration of Stx2 + LPS at day 2 yielded maximal astrocyte reactivity in comparison with control, LPS and Stx2 treated mice (Fig. 3a-h). Also, reactive astrocytes measured by GFAP immunoexpression were significantly increased after 4 and 7 days in Stx2, LPS and Stx2 + LPS mice groups in comparison to the control one. Nevertheless, non significant reactive astrocytes were observed in the expression level of GFAP after 20 days except Stx2 + LPS treated one (Fig. 3j). No immunofluorescence was observed in negative controls by omitting primary antibody (Fig. 3i).

### Stx2 decreased the area occupied by lectin-bound to glycocalyx microvessels and the co-administration with LPS enhanced this effect

**Fig. 4:**
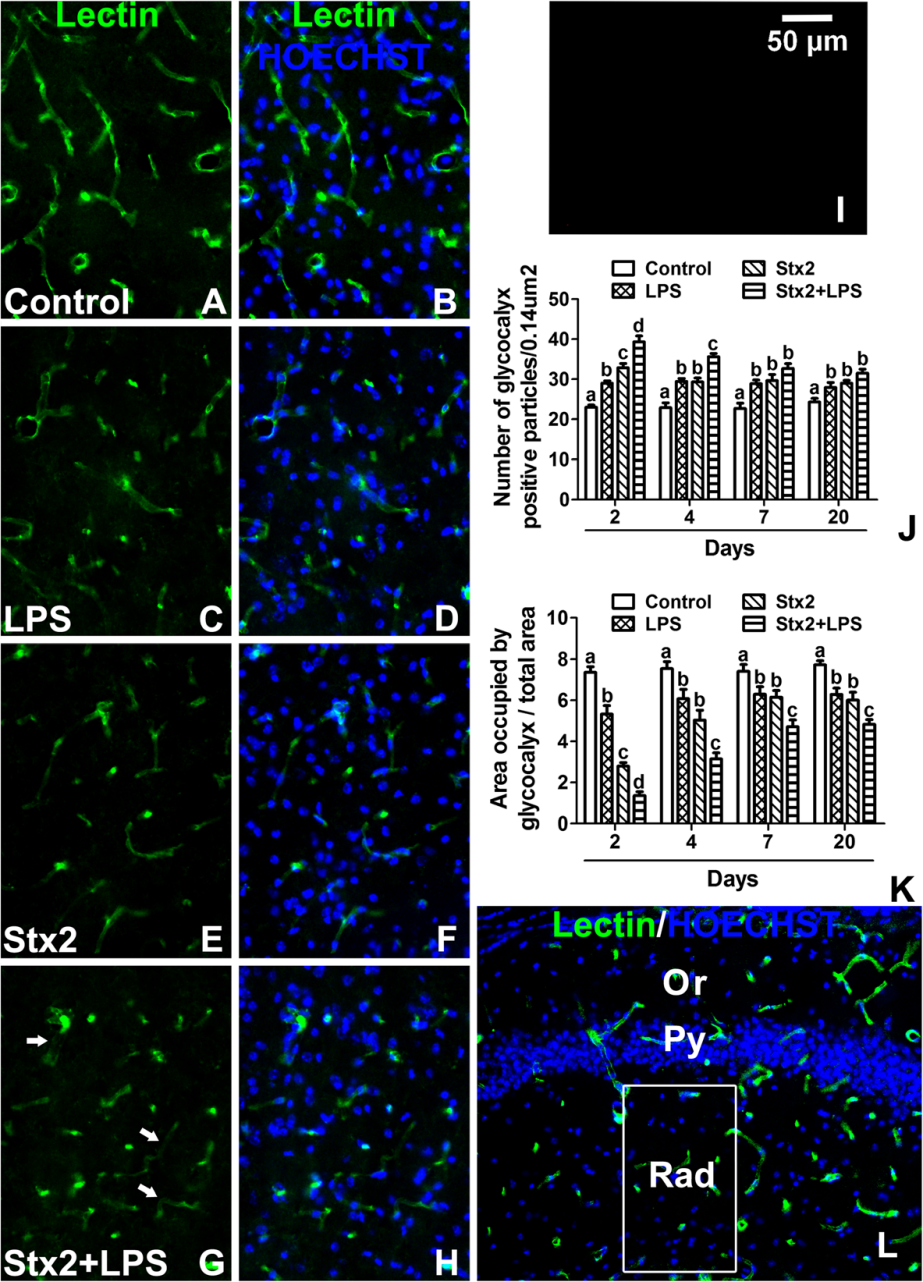
Microvasculature profile of the mouse hippocampus treated with Stx2 or Stx2 + LPS. Control (**a**-**b**), LPS (**c**-**d**), Stx2 (**e**-**f**) and Stx2 + LPS (**g**-**h**) after 2 days of treatments. Representative lectin histofluorescence micrographs (**a**, **c**, **e**, **g**). Merge micrographs between lectin and Hoechst histofluorescences (**b**, **d**, **f**, **h**, **l**). Number of glycocalyx positive particles at 2, 4, 7 and 20 days (**j**). Area occupied by glycocalyx in microvessels from different treatments at 2, 4, 7 and 20 days (**k**). The area analyzed in this study was Rad from the CA1 hippocampal area (drawn rectangle) (**l**). Negative control by omitting a primary antibody (**i**). Different letters (a, b, c, d) above the columns indicate a significant difference between the 4 different i.v.-treated groups (**j**-**k**). The arrows indicate the areas where the lectin-binding glycocalix was not express by endothelial cells (**g**). The scale bar in Fig. I applies to all micrographs. Data were analyzed by One-way ANOVA, Bonferroni’s post hoc test, *p* < 0.05, *n* = 4

Lectins (*Lycopersicum esculentum*) are non-immune proteins that bind with high affinity to specific N-acetyl-D-glucosamine and poly-N-acetyl lactosamine sugar residues of endothelial plasma membrane glycocalyx [20]. Thus, they are useful markers to study the microvasculature profile in the stratum radiatum of the CA1 hippocampal layer (Fig. 4l). Control treated mice (Fig. 4a-b) showed continuous lectin fluorescence binding throughout all microvessels though resulting well preserved, with continuous and defined edges since the 2 days of treatment and persisting at 4, 7 and 20 days of observation (Fig. 4c-h). After 2 days of treatment, the number of microvessels was significantly increased in the LPS, Stx2 and Stx2 + LPS treated mice in comparison to the control one (Fig. 4j). As observed, the maximal number of microvessels expressing the glycocalyx was observed after 2 days in Stx2 + LPS treated hippocampal brain mice. However, the number of these glycocalyx molecules from the different toxin treatments tended in 20 days to match those from the control group (Fig. 4j). According to the area occupied by endothelial glycocalyx, the control treated mice occupied a larger area per observed field than those from the toxins treated mice (Fig. 4k) at day 2, becoming this area minimal when mice were co-treated with Stx2 + LPS (Fig. 4g-h, k). No immunofluorescence was observed in negative controls by omitting primary antibody (Fig. 4i).

### The expression of neuronal NeuN was decreased in hippocampal CA1 pyramidal layer (Py) following the administration of Stx2

**Fig. 5:**
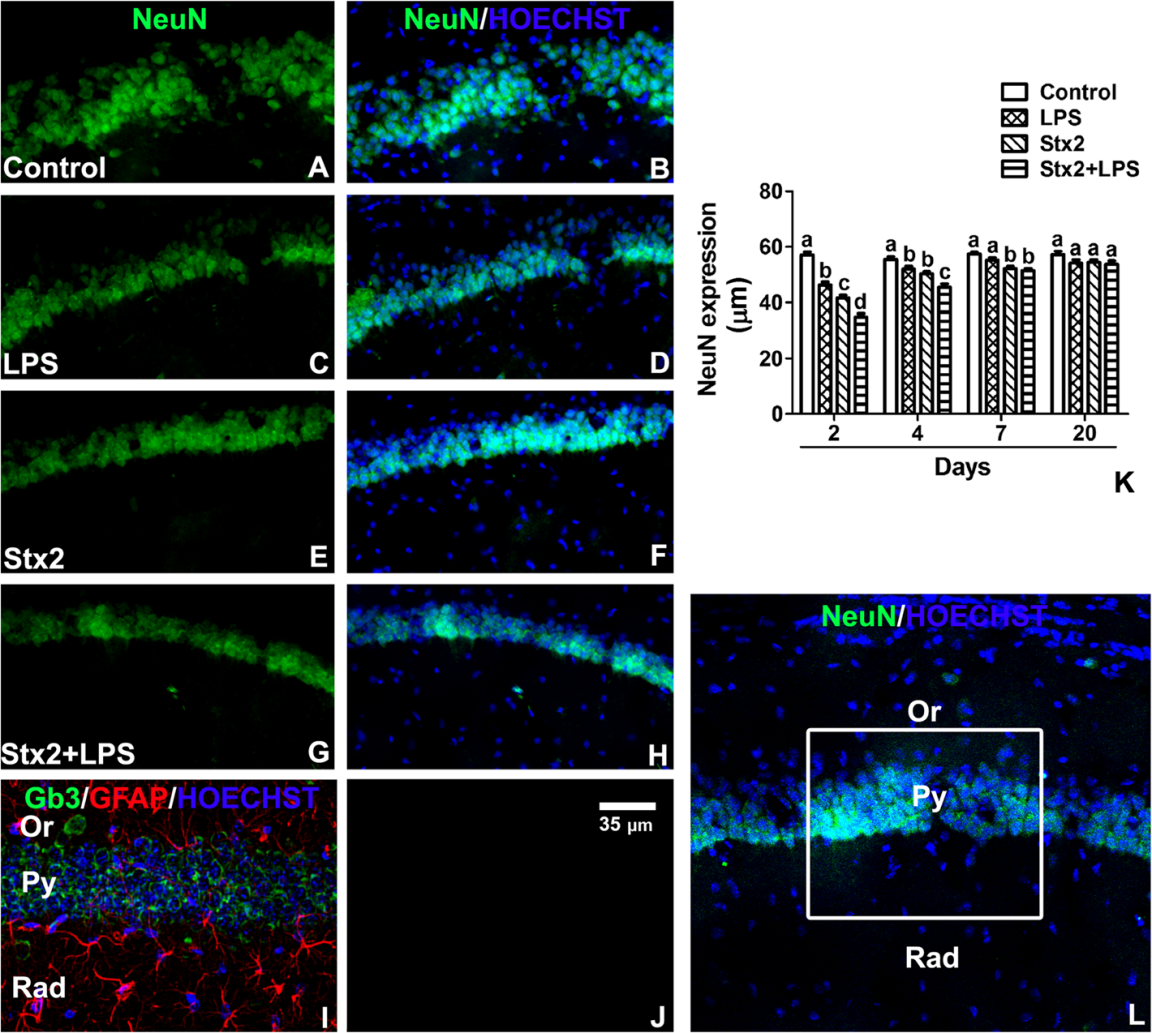
The expression of NeuN from the CA1 Py layer is reduced following toxins treatments. NeuN immunofluorescence (**a**, **c**, **e**, **g**) and merge images between NeuN immunofluorescence and Hoechst histofluorescence (**b**, **d**, **f**, **h**, **l**). Control (**a**-**b**), LPS (**c**-**d**), Stx2 (**e**-**f**) and Stx2 + LPS (**g**-**h**) corresponding to the treatments of day 2. Merge images from a representative hippocampal control section between Gb3, GFAP immunofluorescences and Hoechst histofluorescence (**i**). Negative control by omitting primary antibody (**j**). NeuN expression in CA1 Py layer of all treatments at 2, 4, 7 and 20 days (**k**). Different letters (a, b, c, d) above the columns indicate significant differences between each column (**k**). The drawn rectangle was the area analyzed (**l**). The scale bar in Fig. J applies to all micrographs. Data were analyzed by One-way ANOVA, Bonferroni’s post hoc test, *p* < 0.05, *n* = 4

With the purpose to measure the thickness of the pyramidal layer of neurons from the hippocampus CA1 area (Fig. 5l) an antibody anti-NeuN was employed. NeuN is a splicing alternative factor expressed in neurons and is often used as a neuronal nuclear marker [21, 22]. After 2 days of treatment, the toxins decreased the expression of NeuN that resulted in the reduction of the thickness of Py in comparison with the control. Py was maximally reduced in Stx2 + LPS treated mice (Fig. 5a-h, k). After 4 days of treatment, a decrease in the expression of NeuN was observed in the mice treated with the toxins but in a lesser extent than in those observed at day 2. After 20 days of toxin treatments no significant differences were found in the expression of NeuN and in the thickness of Py (Fig. 5k). Finally, Py neurons immunolabeled with the anti-NeuN antibody were also immunolabeled with an anti-Gb3 antibody (Fig. 5i), indicating that these neurons expressed the Stx2 receptor. No immunofluorescence was observed in negative controls by omitting both antibodies (Fig. 5j).

### The expression of oligodendrocytic MBP myelin protein was decreased following Stx2 administration

**Fig. 6:**
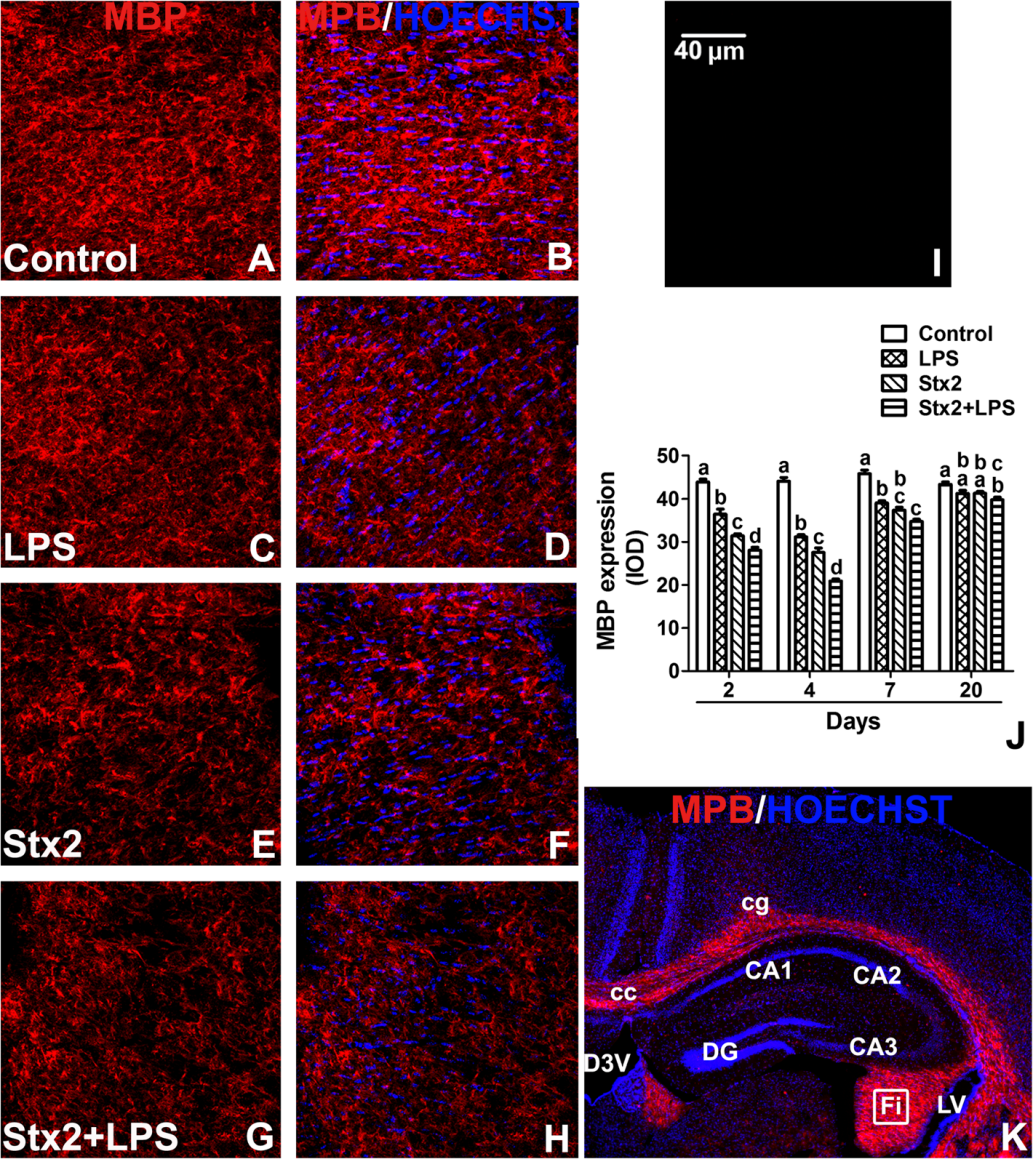
Stx2 and Stx2 + LPS damage the myelin sheath. Micrographs of mouse brain hippocampus after 4 days of treatment with control, LPS, Stx2 or Stx2 + LPS (A-H). Immunofluorescence using an anti-MBP antibody (**a**, **c**, **e**, **g**). Merge images between anti-MBP immunofluorescence and Hoechst histofluorescence (**b**, **d**, **f**, **h**, **k**). Control-treated mice (**a**-**b**); LPS-treated mice (**c**-**d**); Stx2-treated mice (**e**-**f**) and Stx2 + LPS-treated mice (**g**-**h**). Negative control by omitting a primary antibody (**i**). MBP expression under all treatments at 2, 4, 7 and 20 days (**j**). Different letters (a, b, c, d) above the columns indicate a significant difference between each column (**j**). A low magnification micrograph shows different areas of the mouse brain: corpus callosum (cc); dorsal 3rd ventricle (D3V); cingulate cortex (cg); fields of the hippocampus (CA1, CA2, CA3 and DG); lateral ventricle (LV) and fimbria of the hippocampus (Fi); the drawn square in the Fi was the area analyzed (**k**). The scale bar in Fig. I applies to all micrographs. Data were analyzed by One-way ANOVA, Bonferroni’s post hoc test, *p* < 0.05, *n* = 4

In the current murine model, we have previously shown by electron microscopy, among other features, that Stx2 produced disorganized oligodendrocytic myelin sheaths [16]. In the present work, we wanted to determine whether these disorganized myelin sheaths involved also differences in the expression level of myelin basic protein (MBP), a molecule present in mature myelin oligodendrocytes involved in the maturation of the nerves [23], also employed as an oligodrendocytic marker of myelin formation. In the hippocampus, MBP is located in axonal projections of the fornix/fimbria (Fig. 6k). Following Stx2 administration, the expression of MBP significantly decreased after 2 and 4 days (Fig. 6e, f, j). After 7 days the expression of MBP tended to be compensated in comparison to the control, but significant differences between different groups was still observed (Fig. 6j). LPS administration also decreased the expression of MBP in the same days compared with control, however in a lesser extent than Stx2 (Fig. 6c, d, j). Finally, minimal expression of MBP was found following the administration of Stx2 + LPS after 4 days (Fig. 6g, h, j). No immunofluorescence was observed in negative controls by omitting primary antibody (Fig. 6i).

### Stx2 produced microglial reactivity and co-administration of Stx2 with LPS increased this event

**Fig. 7:**
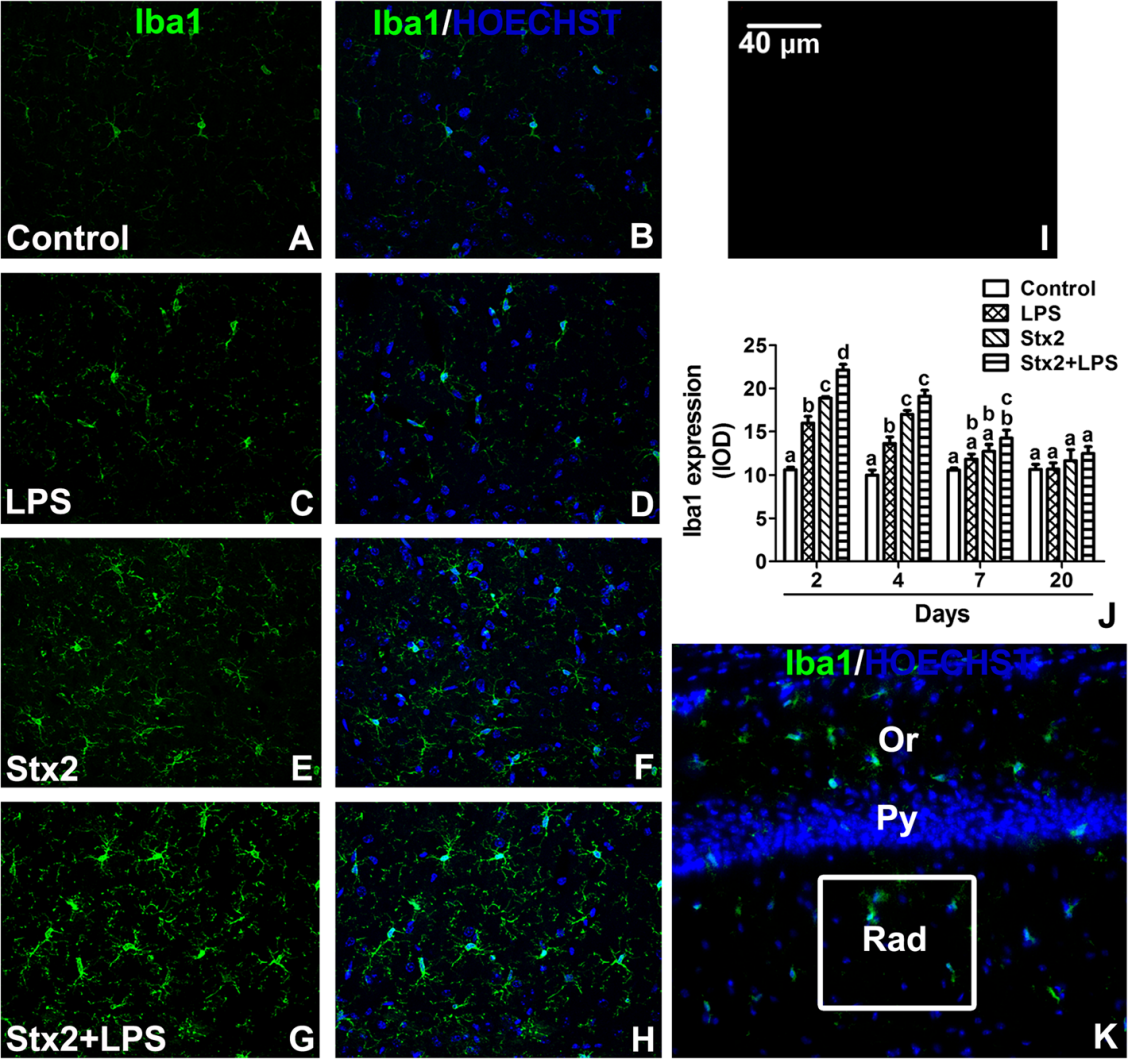
Stx2 and Stx2 + LPS treatments increased the expression level of Iba1. Control (**a**-**b**), LPS (**c**-**d**), Stx2 (**e**-**f**) and Stx2 + LPS (**g**-**h**) after 2 days of treatments. Representative micrographs of anti-Iba1 immunofluorescence (**a**, **c**, **e**, **g**). Merge micrographs between anti-Iba1 immunofluorescence and Hoechst histofluorescence (**b**, **d**, **f**, **h**, **k**). Negative control by omitting a primary antibody (**i**). Iba1 expression levels at 2, 4, 7 and 20 days (**j**). The area analyzed was the drawn square in Rad area from the CA1 hippocampus (**k**). Different letters (a, b, c, d) above the columns indicate significant difference between different groups (**j**). The scale bar in fig. I applies to all micrographs. Data were analyzed by One-way ANOVA, Bonferroni’s post hoc test, *p* < 0.05, *n* = 4

The ionized calcium-binding adaptor molecule 1 protein (Iba1) is a microglia/macrophage-specific calcium-binding protein commonly used to identify microglial activation [24]. Therefore, an anti-Iba1 antibody was used to detect microglia activation by Stx2 and/or LPS in mouse hippocampal stratum radiatum (Fig. 7k). Activated microglial cells were defined by the expression levels of the microglial marker Iba1 (Fig. 7j). Maximal increase in the expression of Iba1 was found in activated microglia after 2 days of co-treatment with Stx2 + LPS, being the treatment with Stx2 + LPS the highest value compared with controls (Fig. 7a-h). Also, significant microglial activation was assessed at day 4 in mice treated with LPS, Stx2 and Stx2 + LPS in comparison to controls, but in a lesser extent compared with day 2 (Fig. 7j). At day 7, only mice treated with Stx2 + LPS displayed significant microglial activation compared to controls (Fig. 7j). Finally, after 20 days, all treated mice with either LPS, Stx2 or Stx2 + LPS showed no significant differences in the expression levels of Iba1 denoting no microglial reactivity, returning to control values. No immunofluorescence was observed in negative controls by omitting primary antibody (Fig. 7i).

### Lipid peroxidation was determined following Stx2 and Stx2 together with LPS administration

**Fig. 8:**
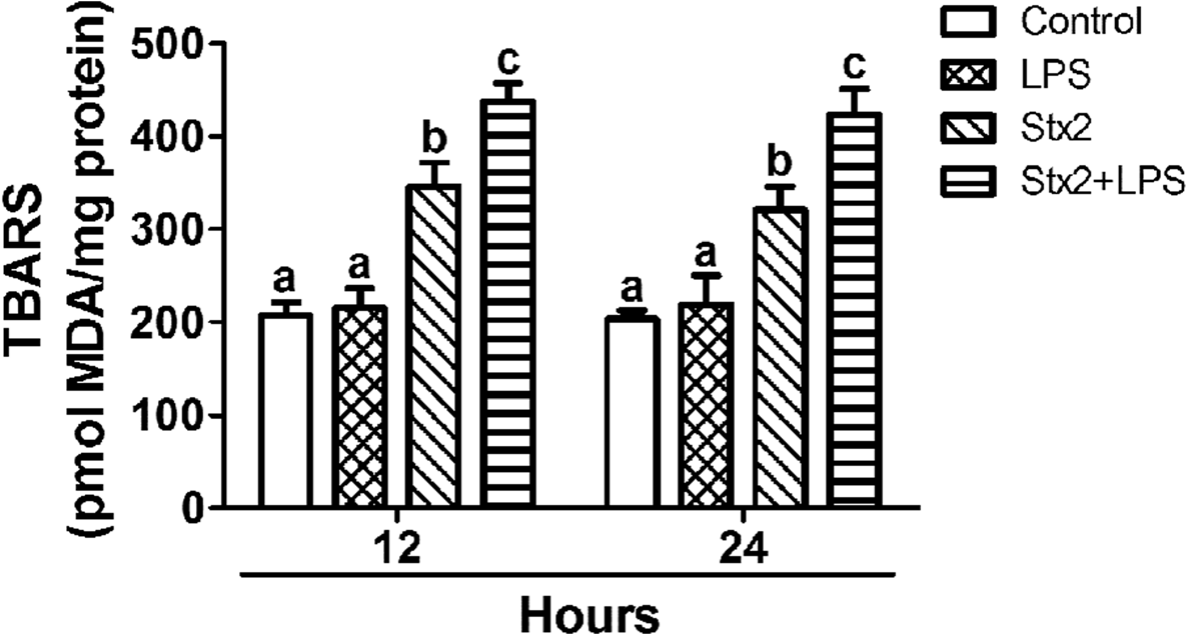
TBARS measurement in the murine hippocampus. The content of lipid peroxidation was measured by Thiobarbituric Acid Reactive Substances (TBARS) after 12 h and 24 h. Data were analyzed by One-way ANOVA, Bonferroni’s post hoc test, *p* < 0.05, *n* = 4

TBARS (thiobarbituric acid reactive substances) assay was determined to evaluate the lipid status in cell membranes of murine hippocampuses (Fig. 8). Malondialdehyde (MDA) content was measured as a marker of lipid peroxidation. After 12 h and 24 h of treatments no significant difference were observed between MDA production in control and LPS treatments. However, MDA content was found significantly increased in Stx2 treated mice respect LPS and control ones, while the treatment with Stx2 + LPS yielded the maximal production of MDA in comparison to all treatments at these periods of time (Fig. 8).

## Discussion

The present results show for the first time the deleterious effects of systemic sub-lethal Stx2 in the hippocampus of murine brains at the cell level. Increased water content in the brain was determined 12 h until 48 h following Stx2 treatment. One possibility that may explain the observed increased in water content is the reported decrease in the expression of aquaporins involved in fluid elimination from the brain and the increased permeability of the BBB leading to brain edema [25]. Stx2 provoked astrocyte reactivity in a dose dependent manner peaking at 48 h. This might have aggravated the observed BBB disarrangement. According with this observation, Stx2 and co-treatment of Stx2 with LPS led to the alteration of the endothelium evidenced by discontinuity of the endothelial glycocalyx which compromised the BBB integrity. As intact glycocalyx is necessary for the maintenance of normal vascular function [26] its discontinuity was reflected as a decrease in the expression of the glycocalyx [27]. This disorder (increased BBB permeability) induced by Stx2 was already demonstrated by our group in the murine brain and cerebellum by using the Evans Blue staining [5, 28]. BBB alteration seemed to impact on CA1 hippocampal neuronal status; as the expression of NeuN was reduced, the thickness of the pyramidal layer was decreased in comparison to controls. Two possibilities may explain the decreased of the thickness in this layer as a consequence of toxin treatments. One possibility is the loss of fluid content in neurons and consequently the reduction of neuronal somatic size, while this was totally reversed after 20 days following the toxins treatments. Neuronal loss of fluid content has been previously reported by us in the murine striatum following the same characterized murine model of HUS-encephalopathy [16]. Alternatively, a temporal inhibition in the expressions of NeuN and MBP might have occurred, which recovered after 20 days [29]. These events could occur in those STEC-HUS patients that resolved acute symptoms from neurological disorders [30]. To confirm this, further experiments would be conducted that escapes the current aim of the present work. In accordance to this, the observed lipid peroxidation may contribute to the reduction in the expression of neuronal NeuN from pyramidal CA1 layer. The assessment of lipid peroxidation has been determined in a model of renal failure following intravenous administration of Stx2 [31]. Then, it was not surprised to detect lipid peroxidation in the hippocampus which may lead to cell death.

Stx2 may target neurons through Gb3 receptor. The presence of this receptor was previously immunolocalized in neurons, but not in astrocytes [14, 32]. Then the possibility that Stx2 may exert a direct neurodegenerative action through Gb3 is highly probable. How Stx2 may target astrocytes then? It was found that Stx2 releases high amounts of glutamate from neurons [32]. This event generates excitotoxicity promoving astrocyte reactivity, an event by which an inflammatory process occurs [33]. Then it could be inferred from the previous description that Stx2 influences astrocytes indirectly. Alternatively, Stx2 may target directly astrocytes not through Gb3 but by the presence of the TLR4 receptor. It has been reported that Stx2 activates neutrophils and release cytokines when it binds the TLR4 receptor [34]. Then, astrocytes may respond in a similar way as neutrophils respond to Stx2.

Microglial reactivity was observed 2 days after toxin treatments. These cells might have contributed to the observed deleterious effects in the CA1 layer. Microglial activation possesses phagocytic properties, but produces and releases detrimental pro-inflammatory cytokines [35]. In addition, reduction of oligodendrocityc MBP may have obeyed as a consequence of the observed astrocytic and microglial activations, and/or neuronal disarrangements. A previous work showed that incubation of Stx2 in cultured oligodendrocytes had no harming effects [36]; however, oligodendrocytes were damaged in a brain context. Then, communication between neurons and oligodendrocytes appears to be essential for healthy myelin [36]. It has been reported that when oligodendrocytes extend its processes they are able to myelinate axons, keep axonal integrity, support axonal metabolism and neuronal survival [37]. On the other hand, microglial cells may damage oligodendrocytes and/or the myelin sheath as they express pro-inflammatory cytokines such as TNFα and/or IL-1β [38, 39]. In addition, they produce ROS and NO radicals, both important sources of oxidative damage observed during the pathogenesis of demyelinating diseases [40]. Lipid peroxidation may occur in oligodendrocytes. In summary, Stx2 reaches the brain affecting the BBB. Edema and lipid oxidation were earlier events. Microglial and/or astrocyte cells could be potential local inducers of the observed lipid damage. According to this, these cells were maximally activated at 2 days following toxin administration which coincided with the observed neuronal alteration (as the thickness of the CA1 pyramidal hippocampal layer decreased). A late event was observed in oligodendrocytic myelin sheath reduction that occurred 4 days following toxins administrations. Further studies should be carried out in order to determine possible mechanisms of cell plasticity observed at 20 days.

## Conclusion

Stx2 damaged the neurovascular unit of the hippocampal area CA1, producing interstitial edema and a decrease in the profile of endothelial-glycocalyx expression that correlated with the disruption of the BBB. Additionally, Stx2 produced astrocytic and microglial cell reactivity and lipid peroxidation. Neurons and oligodendrocytic myelin sheath were found maximally damaged at day 2 and day 4 respectively following Stx2 administration. Co-administration of LPS enhanced the deleterious effect of Stx2 in the hippocampal area CA1. Then, LPS should be taken in consideration in HUS-derived encephalopathy models. The detrimental effects of these toxins were reversed at day 20. The present work could significantly shed light in the understanding of cell mechanisms that lead to hippocampal damage following sub-lethal administration of Stx2 and LPS. This knowledge could impact in the search for alternative treatments in the hippocampus of patients intoxicated with STEC strains.

## Abbreviations

CNS: Central Nervous System
EHEC: Enterohemorrhagic *Escherichia coli*
Gb3: Globotriaosylceramide receptor
GFAP: Glial Fibrillary Acidic Protein
HUS: Hemolytic Uremic Syndrome
i.v.: intravenous
Iba1: Ionized Calcium-Binding Adapter Molecule 1
IOD: Integral Optical Density
LPS: Lipopolysaccharide
MBP: Myelin Basic Protein
MDA: Malondialdehyde
NeuN: Neuronal Nuclear Antigen
STEC: Shiga toxin-producing *Escherichia coli*
Stx2: Shiga toxin 2
TBARS: Thiobarbituric Acid Reactive Substances
